# Outer membrane vesicles from β-lactam-resistant *Escherichia coli* enable the survival of β-lactam-susceptible *E*. *coli* in the presence of β-lactam antibiotics

**DOI:** 10.1038/s41598-018-23656-0

**Published:** 2018-03-29

**Authors:** Si Won Kim, Seong Bin Park, Se Pyeong Im, Jung Seok Lee, Jae Wook Jung, Tae Won Gong, Jassy Mary S. Lazarte, Jaesung Kim, Jong-Su Seo, Jong-Hwan Kim, Jong-Wook Song, Hyun Suk Jung, Gwang Joong Kim, Young Ju Lee, Suk-Kyung Lim, Tae Sung Jung

**Affiliations:** 1 Laboratory of Aquatic Animal Diseases, Institute of Animal Medicine, College of Veterinary Medicine, Gyeongsang National University, Jinju, 52828 Republic of Korea; 2 Department of Animal and Dairy Sciences, Mississippi State University, MS, 39762 USA; 3 Environmental Chemistry Research Center, Korea Institute of Toxicology Gyeongnam Department of Environmental Toxicology and Chemistry, Jinju, 52834 Republic of Korea; 4 Department of Biochemistry, College of Natural Sciences, Kangwon National University, Chuncheon, 24341 Republic of Korea; 5 College of Veterinary Medicine, Kyoungpook National University, Daegu, 41566 Republic of Korea; 6 Bacterial Disease Division, Animal and Plant Quarantine Agency, Gimcheon-si, 39660 Republic of Korea

## Abstract

Outer membrane vesicles (OMVs) containing various bacterial compounds are released from mainly gram-negative bacteria. Secreted OMVs play important roles in the ability of a bacterium to defend itself, and thus contribute to the survival of bacteria in a community. In this study, we collected OMVs from β-lactam antibiotic-resistant *Escherichia coli* established by conjugation assay and the parental β-lactam antibiotic-susceptible strain, and performed comparative proteomic analysis to examine whether these OMVs carried β-lactam-resistant compounds. We also investigated whether both types of OMVs could protect susceptible cells from β-lactam-induced death and/or directly degrade β-lactam antibiotics. Several proteins that can be involved in degrading β-lactam antibiotics were more abundant in OMVs from β-lactam-resistant *E*. *coli*, and thus OMVs from β-lactam resistant *E*. *coli* could directly and dose-dependently degrade β-lactam antibiotics and fully rescue β-lactam-susceptible *E*. *coli* and other bacterial species from β-lactam antibiotic-induced growth inhibition. Taken together, present study demonstrate that OMVs from β-lactam-resistant *E*. *coli* play important roles in survival of antibiotic susceptible bacteria against β-lactam antibiotics. This finding may pave the way for new efforts to combat the current global spread of antibiotic resistances, which is considered to be a significant public health threat.

## Introduction

The discovery of antibiotics has prolonged the human lifespan and aided in the development of various health-related technologies. The efficacies of many antibiotics have been established and improved since the initial identification of penicillin, but the overuse of antibiotics has led to the appearance of “superbugs” as bacteria evolve the means to resist traditional antibiotics. The increased emergence of superbug strains has come to be considered the biggest problem facing public health worldwide. For example, carbapenem-resistant strains of *Acinetobacter baumannii*^1^ and multi-resistant *Pseudomonas aeruginosa*^2^ are currently important clinical problems. In addition, *Staphylococci* strains that are highly resistant to β-lactams (e.g., methicillin-resistant *Staphylococcus aureus*)^2,3^ and *Escherichia coli* and *Klebsiella* species capable of producing extended-spectrum β-lactamases (ESBLs), which confer resistance against β-lactams^2^, are becoming severe problems in antibacterial chemotherapy. The damage caused by antimicrobial resistance has been predicted to reach a total gross domestic product (GDP) loss of 100 trillion USD worldwide by 2050, and resistance could cause 10 million deaths per year by that point^4^. If we hope to prevent the spread of superbugs, we must study all possible mechanisms that bacteria can use to protect themselves against antibiotic agents.

All Gram-negative species, such as *E*. *coli*, release membrane vesicles during *in vitro* growth and *in vivo* infection^5–7^. These spherical membrane bilayer structures originate from the outer membrane, and are thus called Outer Membrane Vesicles (OMVs)^8^. OMVs have average diameters ranging from 10 to 250 nm, and are naturally discharged when part of the bacterial outer membrane bulges out and pinches off^9–11^ via a yet-undefined mechanism. The protein components of OMVs reportedly comprise the expected outer membrane proteins along with lipopolysaccharide, phospholipids, DNA, RNA, cytoplasmic proteins, and periplasmic membrane proteins^5,6,9,12^.

In the past decade, OMVs have been shown to act in various processes, including the formation of biofilm, killing of competing microbes, secretion of bacterial proteins, transmission of virulence and signaling factors, bacterial self-defense, cell-to-cell communication, bacterial pathogenicity, and infection^13–19^. OMVs have also been shown to be involved in organizing group activities and behavior in bacterial populations^15^, as well as transferring proteins and genetic materials^9^. Given their compositions and physicochemical properties, OMVs have been assessed as vaccine candidates against bacterial infections^10,20^. Recently, OMVs have been shown to protect bacteria against several classes of antibiotics, physical stress, chemical stress, and antimicrobial peptides^1,3,12,21^. However, although we know that OMVs possess defensive functions against antibiotics, we know little about the extent and mode of these protective actions. To address the role of OMVs in antibiotic resistance, researchers need to perform proteomic studies followed by functional analyses.

In the present study, we compared the protein compositions of OMVs from β-lactam-resistant *E*. *coli* established by a conjugation assay and the parental β-lactam-susceptible *E*. *coli*, and studied the functional significance of these OMVs in the ability of the β-lactam –susceptible bacteria to survive in the presence of β-lactam antibiotics. Our results suggest that OMVs are essential vehicles that transport important molecules in the extracellular milieu, and that they are required for the growth and survival of the tested bacteria in the presence of β-lactam antibiotics. Our findings may support efforts to address the increasing prevalence of the multidrug-resistant strains that are currently complicating public health worldwide.

## Results

### Comparison of antibiotic resistance patterns

In an effort to compare antibiotic resistance patterns by conjugation assay, we examined the growth inhibition zone of Sal45, RC85, and RC85^+^ using disc diffusion assay with various antibiotics (see Supplementary Table S1). Comparing the diameters of Sal45, RC85, and RC85^+^ growth inhibition zones, the diameters of RC85^+^ appears to be almost the same as Sal45, unlike RC85. Therefore, it was confirmed that the resistance of the antibiotics used in the experiment was transferred from the donor cell (Sal45) to the transconjugant cell (RC85^+^) via the bacterial conjugation assay.

### Physical characterization of OMVs secreted from RC85+ and RC85 cells during *in vitro* growth

**Figure 1:**
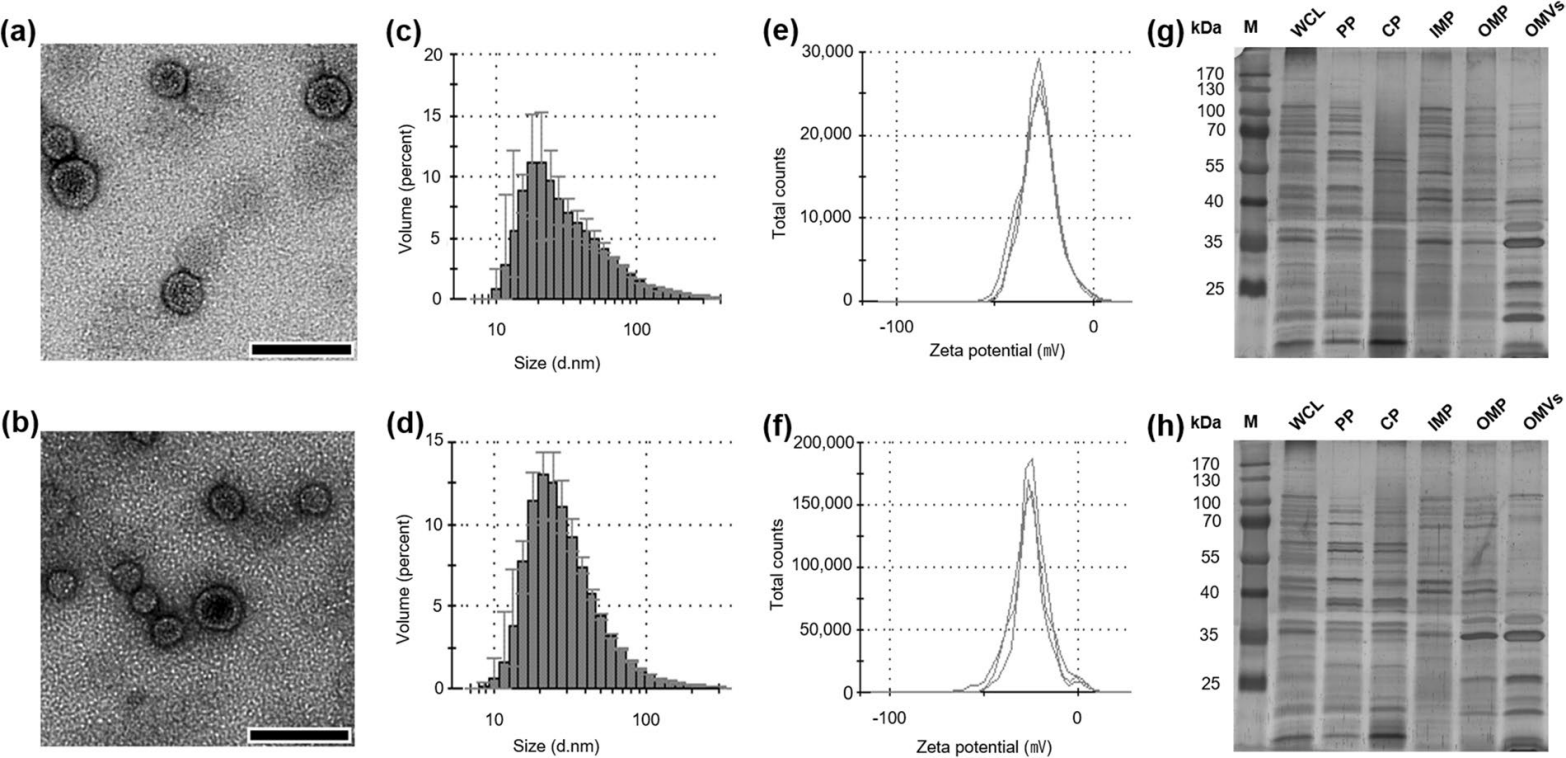
Physical characterization of OMVs secreted from RC85^+^ and RC85 cells. TEM image of OMVs released from RC85^+^ **(a)** and RC85 **(b)** cells. Bar = 100 nm. The size distribution OMVs derived from RC85^+^ **(c)** and RC85 **(d)** cells, as assessed by the Zeta-sizer. Three independent measurements were performed; means are shown with the maximum-minimum (error bars) of the percentage volume. The zeta potential of OMVs from RC85^+^ **(e)** and RC85 **(f)** cells was measured by the Zeta-sizer and each experiment was performed in triplicate. Three micrograms of whole cell lysates (WCLs), periplasmic proteins (PPs), cytoplasmic proteins (CPs), inner membrane proteins (IMPs), outer membrane proteins (OMPs) and OMVs from RC85^+^ **(g)** and RC85 **(h)** cells were separated by 12% SDS-PAGE and then the protein profiles were visualized using silver staining.

To investigate OMVs secreted from RC85^+^ and RC85, OMVs were isolated and examined using transmission electron microscopy (TEM) and dynamic light scattering (DLS). The TEM images of RC85^+^ and RC85 OMVs revealed bi-layered spherical vesicles with an approximate size range of 10 to 50 nm (Fig. 1a,b). The average diameter of the RC85^+^ OMVs was slightly bigger than that of the RC85 OMVs (86.19 ± 0.49 nm vs. 66.90 ± 0.71 nm, respectively; Fig. 1c,d, and see Supplementary Table S2). Their polydispersity factors were 0.269 ± 0.007 and 0.266 ± 0.006, respectively (Table S2), indicating that the preparations were monodispersed. The zeta potentials were −26.87 ± 1.24 mV and −26.77 ± 1.68 mV, respectively (Fig. 1e,f, and Table S2), suggesting that there was no significant difference in the stability of the particles. To examine whether the vesicles carried bacterial proteins, purified OMVs and other purified bacterial component proteins were subjected to SDS-PAGE and compared using the silver staining method (Fig. 1g,h). Comparison of the protein profiles of the WCL, PP, CP, IMP, OMP, and OMV fractions clearly showed that some proteins from each of these bacterial components could be found in the OMVs. In particular, the OMV proteins that formed thick bands in the gel were highly similar to those of the OMP fraction. Equal amounts of OMVs were separated by 12% (w/v) SDS-PAGE (see Supplementary Fig. S1) to compare the protein components of OMVs from RC85^+^ and RC85 cells by LC-ESI-MS/MS.

### OMVs from antibiotic-resistant *E*. *coli* can protect susceptible cells against β-lactam antibiotics

**Figure 2:**
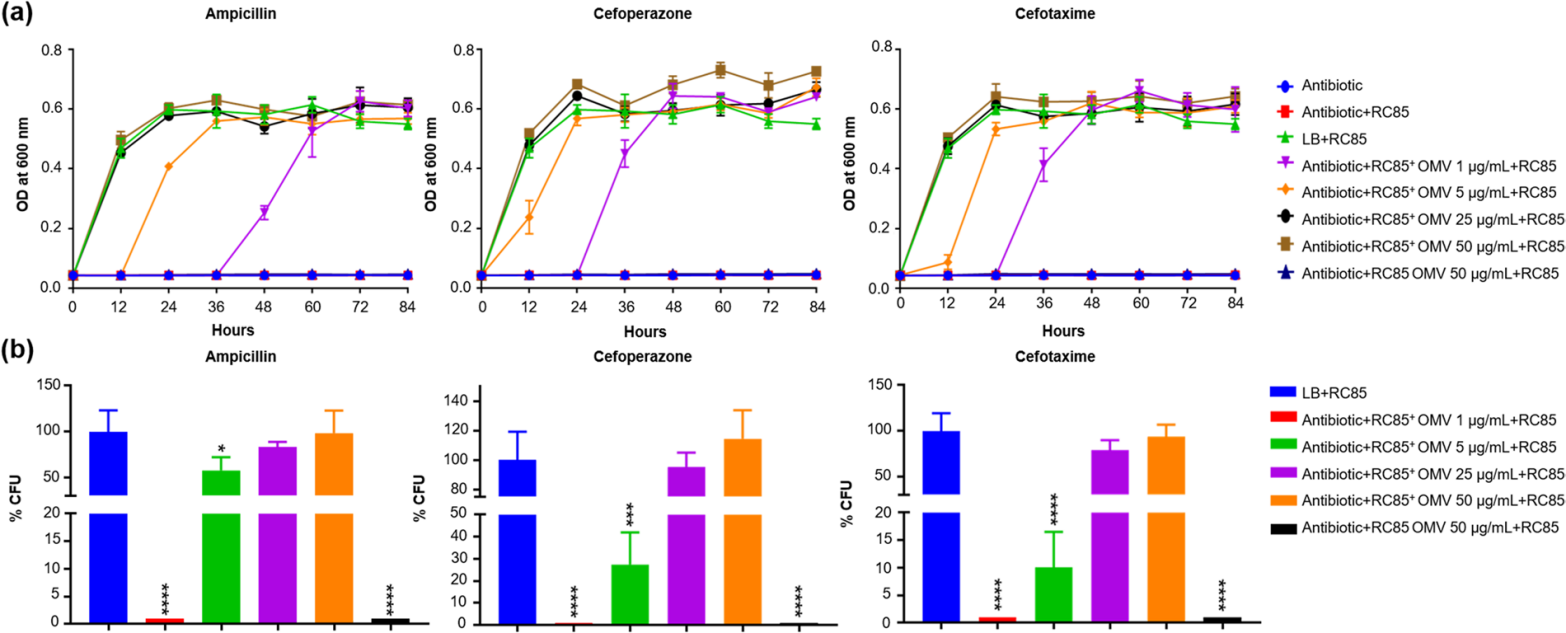
OMVs from β-lactam-resistant *Escherichia coli* can protect β-lactam-susceptible *Escherichia coli* and fully rescue them from β-lactam antibiotic-induced growth inhibition. (**a**) Representative growth profiles of RC85 cells in the presence of growth-inhibiting concentrations of antibiotics. The growth-inhibiting concentrations of antibiotics were: ampicillin, 30 μg/mL; cefoperazone, 4 μg/mL; and cefotaxime, 1.25 μg/mL. The data are presented as means and SEMs of at least three independent experiments. (**b**) The survival percentages of RC85 cells in the presence of the above-listed growth-inhibiting concentrations of antibiotics and RC85^+^ OMVs or RC85 OMVs were computed by bacterial counts of cultures at a certain time points (ampicillin, 12 h; cefoperazone and cefotaxime, 24 h). The data are presented as means and SEMs of three independent experiments. *P < 0.05, **P < 0.01, ***P < 0.001, and ****P < 0.0001.

To determine the effect of different concentrations of OMVs on the growth of antimicrobial agent-susceptible *E*. *coli* (RC85) cells in the presence of growth-inhibiting concentrations of three β-lactam antibiotics, we evaluated the minimum inhibitory concentrations (MICs) of RC85^+^ and RC85 cells against these antibiotics (Table 1). The growth kinetics presented in Fig. 2a show that RC85^+^ OMVs, but not RC85 OMVs, dose-dependently increased the survival of RC85 cells in the presence of all tested antibiotics, compared to controls. Similar growth was seen among RC85 cells cultured without antibiotics as positive control, and those cultured with 25 and 50 μg/mL RC85^+^ OMVs alone. In terms of dosage effects, 1 μg/mL of RC85^+^ OMVs improved the growth of RC85 cells in each of the three antibiotics, whereas 50 μg/mL OMVs from RC85 cells had no such effect over 84 h of incubation (Fig. 2a).

**Table 1:** Minimum inhibitory concentrations (MICs) of three antibiotics against the tested bacteria.

Isolate	MIC (ug/mL)		
	Ampicillin	Cefoperazone	Cefotaxime
RC85	8	≤1/4	≤1/4
RC85^+^	>256	>256	>256

After 84 h, all samples were streaked on TSA agar with and without antibiotics to investigate if the observed growth of RC85 cells was due to OMV-mediated protection or the transfer of antibiotic resistance genes from RC85^+^ OMVs to RC85 cells. All of the tested samples grew on TSA agar without antibiotics but were unable to grow on TSA agar containing the respective tested antibiotics (data not shown). This suggests that RC85^+^ OMVs did not deliver genes related to resistance against ampicillin, cefoperazone, or cefotaxime to RC85 cells, but rather possess some substances that can concentration-dependently protect RC85 cells against the three tested antibiotics.

### Percentage of protection by OMVs

To determine the extent of the OMV-mediated protection of RC85 cells against antibiotics and compare the protective properties of RC85^+^ OMVs and RC85 OMVs, we carried out quantitative plate assays based on growth kinetics. We found that RC85 cells treated with RC85^+^ OMVs grew in the presence of each antibiotic, with the percentage of surviving RC85 cells varying with the antibiotic, dose, and incubation time (Fig. 2b). Cultures treated with 25 and 50 μg/mL RC85^+^ OMVs contained surviving RC85 cells in the presence of ampicillin at 24 h, and cefoperaonze and cefotaxime at 12 h. Surviving RC85 cells were recovered from cultures treated with 5 μg/mL RC85^+^ OMVs at 12 h for cefoperazone and cefotaxime, and at 24 h for ampicillin (Fig. 2b). Moreover, surviving RC85 cells were recovered from cultures treated with 1 μg/mL RC85^+^ OMVs at 36 h for cefoperazone and cefotaxime, and at 48 h for ampicillin (data not shown). However, no surviving cell was recovered from RC85 cell cultures treated with 50 μg/mL RC85 OMVs and incubated for 84 h in the presence of the various antibiotics (data not shown). The above finding are consistent with the results of our growth curve experiments (Fig. 2a) in showing that the amount of RC85^+^ OMVs required to protect RC85 cells may differ according to the applied antibiotic, and that RC85 OMVs cannot protect RC85 cells from the three tested antibiotics. To rule out contamination, colonies obtained from each quantitative plate assay were randomly chosen (*n* = 5, per plate). Indeed, all were confirmed as *E*. *coli* at the species level by MALDI-TOF MS (see Dataset 1).

### OMVs from antimicrobial agent-resistant *E*. *coli* enable antimicrobial agent-susceptible bacteria to survive antibiotic treatment

**Figure 3:**
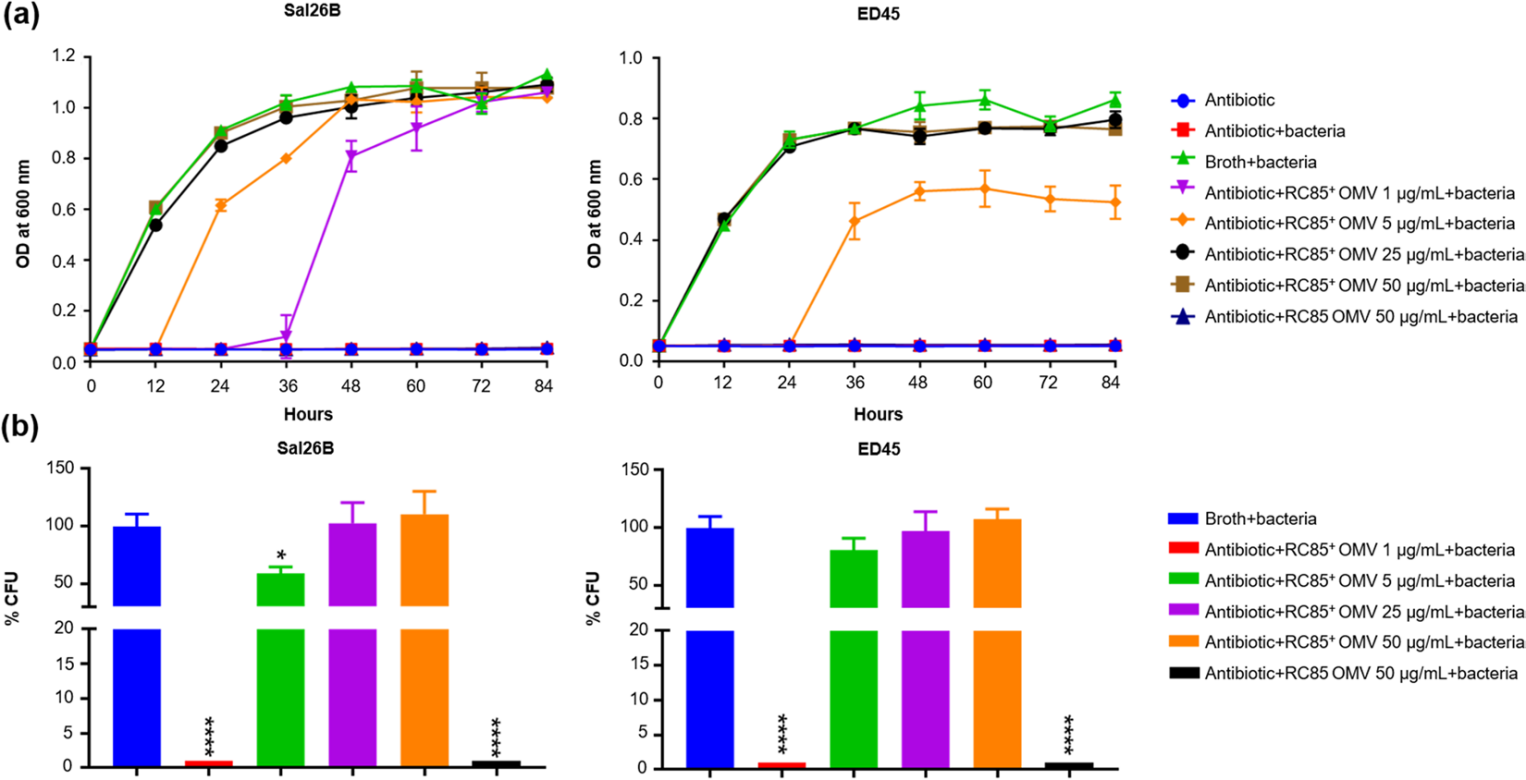
OMVs from β-lactam-resistant *Escherichia coli* defense other bacterial species from β-lactam antibiotic-induced growth inhibition. (**a**) Representative growth profiles of Sal26B and ED45 cells in the presence of a growth-inhibiting concentration of ampicillin (30 μg/mL) plus increasing amounts of RC85^+^ or RC85 OMVs. The data are presented as means and SEMs of at least three independent experiments. (**b**) The survival percentages of Sal26B and ED45 cells were calculated by counting CFUs at specific time points (Sal26B, 24 h; ED45, 36 h) from cultures grown with 30 μg/mL ampicillin and increasing quantities of RC85^+^ or RC85 OMVs. The data are presented as means and SEMs of three independent experiments. *P < 0.05, **P < 0.01, ***P < 0.001, and ****P < 0.0001.

To investigate whether the protective properties of OMVs can affect bacteria of a different genus, we evaluated growth curve profiles and performed qualitative plate assays. *Salmonella* spp. (Sal26B) and *Edwardsiella tarda* (ED45) were incubated with OMVs from *E*. *coli* RC85^+^ and RC85 OMVs in the presence of a growth-inhibiting concentration of ampicillin, and the OD_600_ was measured every 12 h for 84 h. The MICs of ampicillin against Sal26B and ED45 cells were 8 μg/mL and 4 μg/mL, respectively (data not shown). In the presence of RC85^+^ OMVs, Sal26B and ED45 cells both exhibited exponential growth in ampicillin-containing medium in an OMV dose-dependent manner (Fig. 3a). Sal26B cells supplemented with 1 μg/mL RC85^+^ OMVs exhibited growth at 36 h, whereas ED45 cells treated with the same concentration of RC85^+^ OMVs failed to grow by 84 h. ED45 cells supplemented with 5 μg/mL RC85^+^ OMVs could grow at 36 h. Neither bacterial strain grew in ampicillin-containing medium when supplemented with RC85 OMVs, even at a concentration of 50 μg/mL RC85 OMVs (Fig. 3a). After 84 h, all of the samples were streaked on BHI or TSB agar with or without 30 μg/mL ampicillin. All of the samples could grow on nutrient agar without ampicillin, but failed to grow on ampicillin-containing agar (data not shown). The percentages of survivors were calculated by counting the CFUs obtained from growth kinetic experiments. The results revealed that RC85 OMVs did not protect Sal26B or ED45 against ampicillin, whereas RC85^+^ OMVs protected both bacteria depending on dose of OMVs (Fig. 3b). The colonies on the agar plates were correctly identified as *Salmonella* spp. and *Edwardsiella tarda* at the genus and species levels, respectively, by MALDI-TOF MS (Dataset 1), indicating that the culture media were not contaminated.

Together, our findings suggest that susceptible bacteria can grow in the presence of a growth-inhibiting concentration of ampicillin when supplemented with RC85^+^ OMVs, and that this does not reflect the transfer of ampicillin resistance genes, but rather the protective effects of the OMVs. In addition, our observations demonstrate that the ability of RC85^+^ OMVs to protect bacteria against ampicillin is not confined to the same species, but can also be seen (to varying degrees) with bacteria of other genera.

### Degradation of β-lactam antibiotics by OMVs

**Figure 4:**
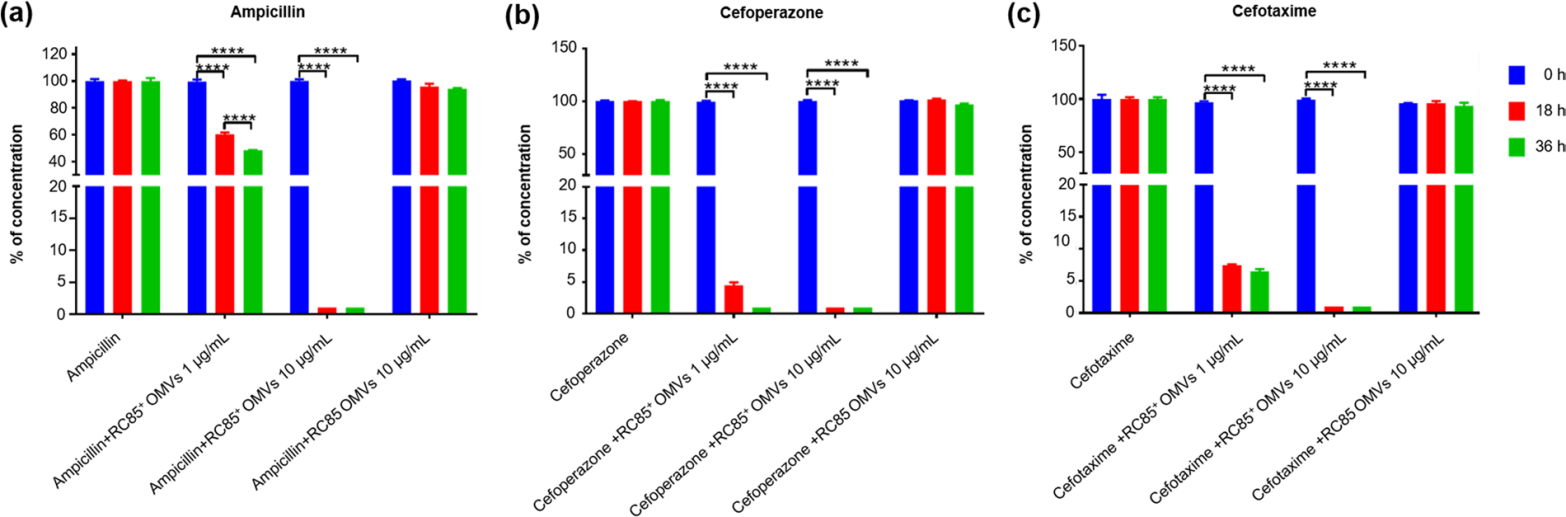
LC-QQQ-based assessment of the concentration of antibiotics following incubation with different doses of RC85^+^ or RC85 OMVs in a cell-free system. The initial concentrations were as follows: ampicillin, 20 μg/mL (**a**); cefoperazone, 5 μg/mL (**b**); and cefotaxime, 5 μg/mL (**c**). One microgram per milliliter or 10 μg/mL of RC85^+^ OMVs or 10 μg/mL of RC85 OMVs in sterilized PBS were mixed with ampicillin, cefoperazone, or cefotaxime. Filtered PBS containing the respective antibiotics without OMVs were used as a positive control and taken as 100%, and the corresponding concentrations of antibiotics in samples treated with RC85^+^ OMVs or RC85 OMVs were analyzed. All samples were incubated at 37 °C with shaking at 150 rpm, and diluted 20-fold for measurements. The concentrations of antibiotics were recorded at 18-h intervals for 36 h in triplicate. Bars indicate standard deviations. ****P < 0.0001.

To determine whether the tested antibiotics could be degraded by the OMVs, LC-ESI-QQQ analysis was performed to measure the concentration of antibiotics after 18 and 36 h of incubation of OMVs in a cell-free system. The concentrations of antibiotics in OMV-free experiments were taken as 100%, and the corresponding concentrations of antibiotics in samples treated with RC85^+^ OMVs or RC85 OMVs were analyzed (Fig. 4). A significant difference was observed between measurements of concentrations of antibiotics treated with OMVs from RC85^+^ and handled with OMVs from RC85 when they were compared with antibiotics without OMVs. The concentrations of ampicillin, cefoperazone, and cefotaxime were significantly decreased in samples treated with 10 μg/mL RC85^+^ OMVs at 18 h. As little as 1 μg/mL of RC85^+^ OMVs degraded 5 μg/mL of cefoperazone or cefotaxime to 10% of their initial concentrations at 18 h, and degraded 20 μg/mL ampicillin to less than 50% of its initial concentration at 36 h. In contrast, 10 μg/mL of RC85 OMVs failed to decrease the concentration of antibiotics at 36 h. Thus, OMVs from RC85^+^ cells can degrade the three tested β-lactam antibiotics to different degrees, whereas those from RC85 show no ability to degrade these antibiotics.

### Identifying the protective substances of RC85^+^ OMVs

**Figure 5:**
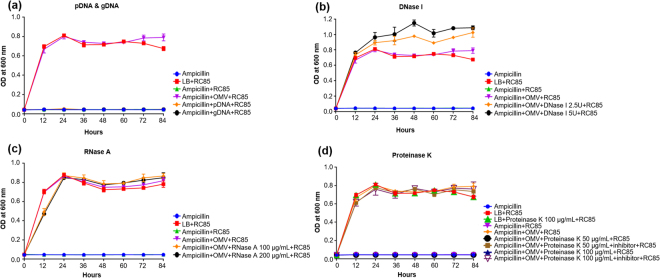
Growth kinetic profiles of RC85 cells in the presence of a growth-inhibiting concentration of ampicillin (30 μg/mL) plus 15 μg/mL of RC85^+^ OMVs pretreated with various enzymes, or with plasmid or genomic DNA instead of OMVs. The treatments included: 2.5 μg of pDNA or gDNA (**a**); 2.5 or 5 U of DNase I (**b**); 100 or 200 μg/mL of RNase A (**c**); and 50 or 100 μg/mL of proteinase K with or without a protease inhibitor cocktail (**d**). The data are presented as means and SEMs of three independent experiments.

To determine which component(s) of RC85^+^ OMVs contribute to their protective effects, we exposed RC85 cells to a growth-inhibiting concentration of ampicillin plus: 1) OMVs that had been pre-treated with various enzymes; or 2) pDNA and gDNA extracted from RC85^+^ cells. We then performed growth kinetic analyses (Fig. 5). Our results revealed that RC85 cells treated with pDNA or gDNA were unable to grow in the presence of ampicillin (Fig. 5a), indicating that DNA is not involved in the ability of RC85^+^ OMVs to protect against ampicillin, and the antibiotic resistance of RC85^+^ cells could not be delivered to RC85 cells in this manner. In these experiments, cells were treated with more pDNA or gDNA than found in 30 μg of OMVs (data not shown). In the enzyme experiments, we found that RC85 cells treated with DNase I-pretreated RC85^+^ OMVs showed increased growth relative to those treated with non-pretreated RC85^+^ OMVs (Fig. 5b). This might be because of the presence of 20 mM MgCl_2_ in the 10X reaction buffer for DNase I in the setup, which might induce an increase in the relative maximum growth rate of *E*. *coli*^22^. Actually, the maximum growth rate of RC85 and RC85^+^ cells in the DNase I reaction buffer-treated group was found to increase when compared with the untreated group (data not shown). Furthermore, RC85 cells cultured with RNase A-pretreated RC85^+^ OMVs showed a growth performance nearly identical to that of RC85 cells cultured with non-pretreated RC85^+^ OMVs (Fig. 5c). Lastly, RC85 cells incubated with proteinase K-pretreated RC85^+^ OMVs failed to grow in the presence of ampicillin through the 84-h experimental period, whereas those cultured with RC85^+^ OMVs that had been exposed to proteinase K plus a proteinase inhibitor cocktail grew comparably to RC85 cells treated with non-pretreated RC85^+^ OMVs (Fig. 5d). Thus, the released free DNA and free RNA released from vesicle preparations do not appear to be involved in their protective effects, whereas the strong effect of proteinase K suggests that proteins mediate the ability of RC85^+^ OMVs to protect bacteria against antibiotics.

### Proteomic analysis of OMVs from RC85^+^ and RC85 cells

**Figure 6:**
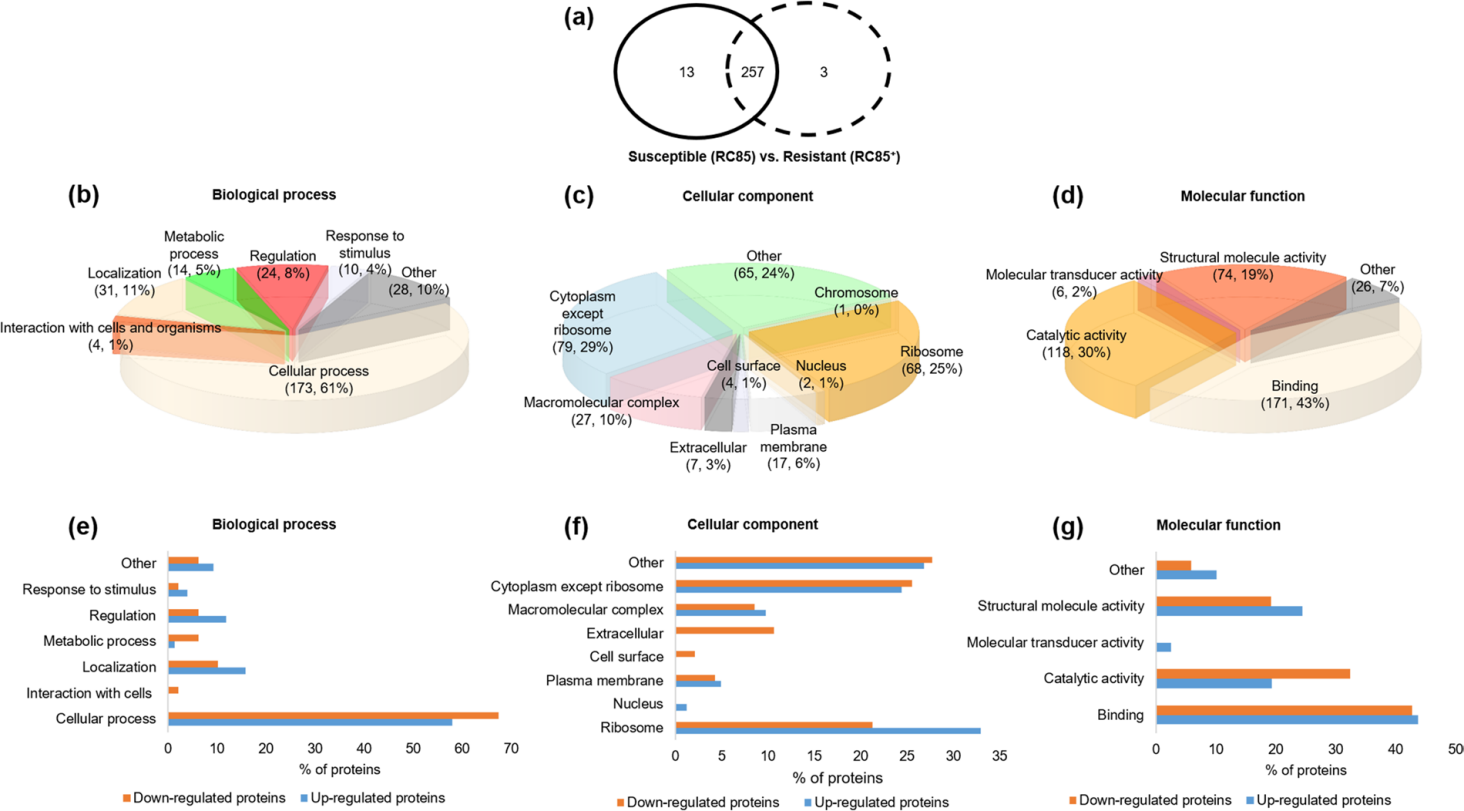
Venn diagrams show the total proteins obtained from RC85^+^ and RC85 OMVs while the bar graphs categorize the proteins that showed differential expression in RC85^+^ OMVs vs. RC85 OMVs. (**a**) A total of 273 proteins were identified in RC85^+^ OMVs (260 proteins) and RC85 OMVs (270 proteins) together. These total proteins were categorized based on the related biological process (**b**), cellular component (**c**), and molecular function (**d**). The numbers of upregulated (red) and downregulated (blue) proteins were compared with respect to the relevant biological process (**e**), cellular component (**f**), and molecular function (**g**).

In an effort to identify the proteins associated with the ability of RC85^+^ OMVs to degrade antibiotics, we compared the protein components of OMVs from RC85^+^ and RC85 cells. A total of 30276 MS/MS spectra were obtained using LC-MS/MS once. We identified 1,693 peptides from the OMVs of RC85^+^ and RC85 cells by a combined dataset. To identify proteins that might be differentially expressed between RC85^+^ OMVs and RC85 OMVs, we discarded any protein that was identified by only one peptide, and those with less than a two-fold intensity difference (indicating up- or down-regulation) in RC85^+^ OMVs versus RC85 OMVs. Overall, 273 individual proteins were mapped; of them, 260 were found in RC85^+^ OMVs, while 270 were found in RC85 OMVs (Fig. 6; see Dataset 2–1). The total proteins of OMVs from RC85^+^ and RC85 and total differentially expressed proteins between them were classified according to the relevant biological processes, cellular components, and molecular functions (Fig. 6). Of the total OMV proteins from RC85^+^ and RC85 related to biological processes, cellular process proteins (*n* = 173) were the most abundant, followed by localization proteins (*n* = 31), and other proteins (*n* = 28) (Fig. 6b). Regarding cellular components, 79 of the total OMV proteins were cytoplasmic proteins except for ribosome and 68 were ribosomal proteins (Fig. 6c). Sixty-five proteins were categorized as “other” proteins, and included components of the cell periphery, membrane parts, periplasmic space, envelope, intracellular proteins, cell projections, and others (Fig. 6c; see Dataset 3–1). Of them, the groups of cell periphery and membrane parts included the outer membrane proteins (44/65, 67.7%). With respect to molecular functions, the total proteins of OMVs from RC85^+^ and RC85 cells included 171 proteins associated with binding, 118 associated with catalytic activity, and 74 associated with structural molecule activity (Fig. 6d).

Eighty-three proteins were upregulated and 49 proteins were downregulated in RC85^+^ OMVs versus RC85 OMVs (Dataset 2–2 and Dataset 2–3). The proteins of these subgroups were also categorized with respect to their biological processes, cellular processes, and molecular functions (Fig. 6e–g). Among all differentially expressed proteins, the cellular process component dominated, followed by localization-related proteins in the biological process perspective (Fig. 6e). In the cellular component group (Fig. 6f), further analysis of the proteins of the “other” group revealed that 20/22 (90.9%) up-regulated proteins and 6/13 (46.2%) down-regulated proteins of this group were predicted to be positioned in the outer membrane (Dataset 3–2 and Dataset 3–3). In the molecular function group, proteins associated with binding function were the most abundant in both up- and downregulated OMV proteins (Fig. 6g).

### Comparative analysis of OMV proteins related to resistance of antibiotics or antimicrobial peptides

The analyzed proteins of OMVs from RC85^+^ and RC85 cells included various proteins expected to be involved in the resistance to β-lactam antibiotics, such as Blc1^3,12,23,24^, OmpC^25–27^, OmpF^25,28^, OmpW^25–27^ and TolC^25–27^ (see Dataset 4). The β-lactamase, CTX-M-1 (also called Blc1, which will be used herein), can inactivate β-lactam antibiotics and was previously identified in OMVs from other bacterial strains^3,12,23,24,29^. OmpW, OmpC, OmpF, and Blc1 were elevated by 2.28- to 19.68-fold, whereas TolC was decreased by 3.56-fold when compared the protein compositions of RC85^+^ OMVs versus RC85 OMVs (Dataset 4). The identified proteins also included some whose putative roles are related to other antibiotics or antimicrobial peptides, including FadL^25^, LamB^25–27,30^, MipA^31^, OmpA^27,32^, OmpT^7,33^, and Tsx^25–27^ (see Dataset 5). FadL and LamB was decreased by 12.62- to 21.44-fold, whereas MipA, OmpA, OmpT, and Tsx were increased by 3.9- to 23.6-fold when compared the protein compositions of RC85^+^ OMVs versus RC85 OMVs (Dataset 5).

### Detection and quantification of β-lactamase activity

**Figure 7:**
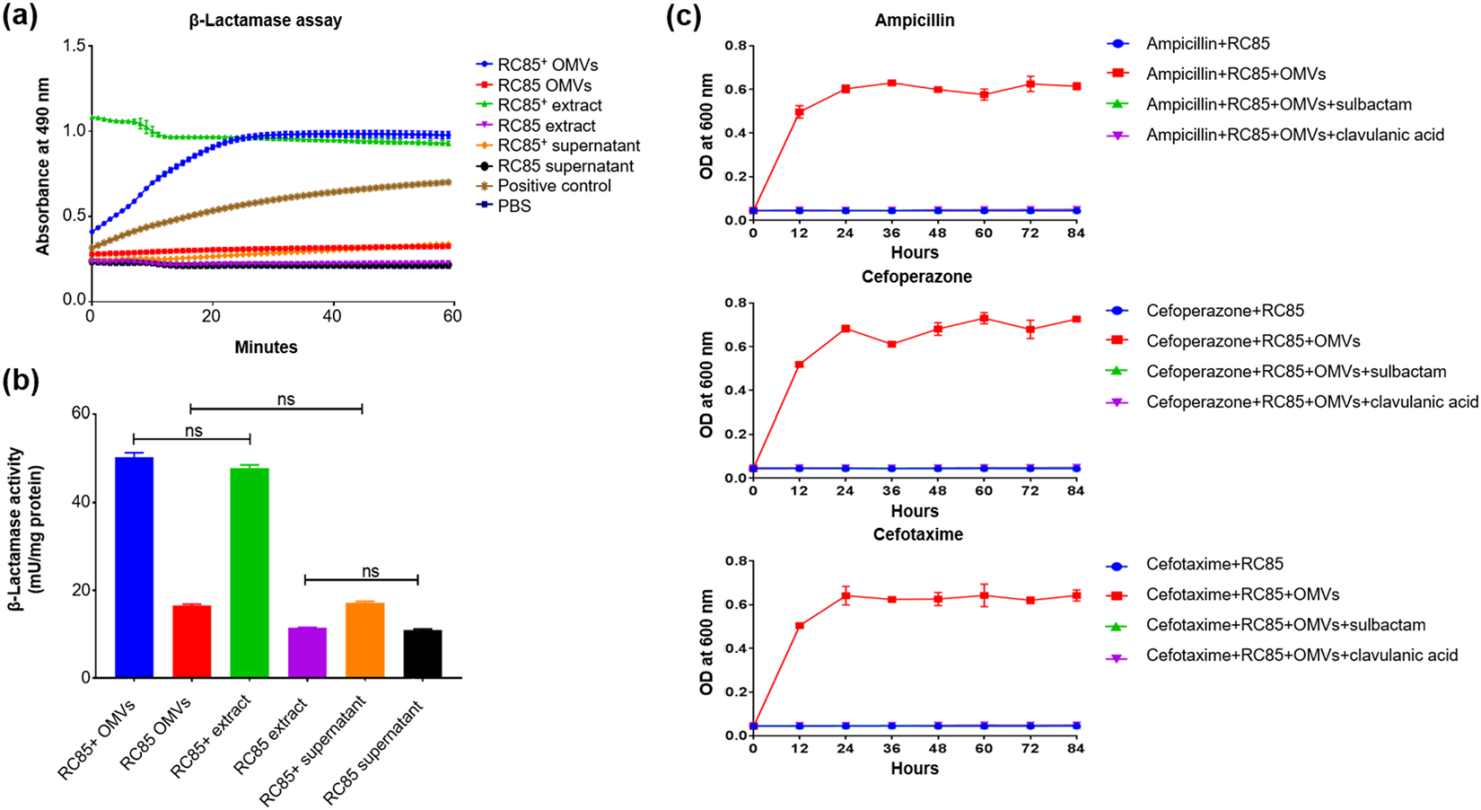
Investigation of the differences in β-lactamase activity between cell extracts, culture supernatant, and OMVs from RC85^+^ and RC85 cells. (**a**) β-Lactamase activity profiles of respective samples, as obtained by measuring absorbance at 490 nm in kinetic mode. The data are presented as means and SEMs of three independent experiments. (**b**) β-Lactamase activities expressed per milligram of protein. One way ANOVA and Tukey’s test were used for analyses, and the data are presented as means and SEMs of three independent experiments. The abbreviation ‘ns’ means not significant. (**c**) The effects of the β-lactamase inhibitors, clavulanic acid and sulbactam, were investigated by growth curve experiments of RC85 cells treated with antibiotics (ampicillin, 30 μg/mL; cefoperazone, 4 μg/mL; cefotaxime, 1.25 μg/mL) plus clavulanic acid or sulbactam in the presence of 50 μg/mL RC85^+^ OMVs. OD_600_ was recorded every 12 h for 84 h from three independent batches.

We used a β-lactamase activity assay kit to investigate the differences in β-lactamase activity between cell extracts of RC85^+^ and RC85 cells, supernatants from RC85^+^ and RC85 cell cultures, and OMVs from RC85^+^ and RC85 cells over time (Fig. 7). The β-lactamase activities of these samples were compared to those of the negative control (PBS) and positive control based on OD_490_ measurements. The absorbances of the RC85^+^ OMVs and crude RC85^+^ cell extracts were higher than those of the positive control at all sampled time points during 60 min (Fig. 7a). The β-lactamase activity of RC85^+^ cells was saturated immediately after the reaction began, whereas that of RC85^+^ OMVs was saturated within 30 min. The cell extracts and culture supernatants of RC85 cells exhibited the same β-lactamase activity as the negative control. The RC85^+^ cell supernatant and RC85 OMVs had β-lactamase activities that were lower than that of the positive control but slightly higher than that of the negative control. The β-lactamase activity of each sample is indicated per milligram of protein (Fig. 7b) and statistical data are presented in Supplementary Table S3. Crude cell extracts of RC85^+^ cells and OMVs derived from RC85^+^ had maximum β-lactamase activities of 50.3 mU/mg and 47.9 mU/mg, respectively. In general, each RC85^+^ sample exhibited more β-lactamase activity than the corresponding RC85 sample, with a 4.2-fold difference seen for cell extracts and a 3.1-fold difference seen for the OMVs.

### Function of OMVs mixed with a β-lactamase inhibitor

To examine whether the β-lactamase activity of RC85^+^ OMVs can degrade β-lactam antibiotics, we measured the growth kinetics of RC85 cells in broth containing β-lactam antibiotics, RC85^+^ OMVs, and a β-lactamase inhibitor (clavulanic acid or sulbactam). Indeed, whereas RC85 could grow in the presence of RC85^+^ OMVs and β-lactam antibiotics, these cells could not survive in the co-presence of antibiotics, RC85^+^ OMVs, and clavulanic acid or sulbactam (Fig. 7c). This finding indicates that RC85^+^ OMVs can degrade β-lactam antibiotics via their β-lactamase activity, and that this activity/function can be blocked by a β-lactamase inhibitor.

## Discussion

In this study, we generated β-lactam-resistant *E*. *coli* (RC85^+^) by conjugation assay using multidrug-resistant *Salmonella sp*. and antimicrobial-sensitive *E*. *coli* (RC85). After that we isolated OMVs from RC85^+^ and RC85 cells (i.e., same species of *E*. *coli*, differing only in their antibiotic resistance), examined their ability to confer resistance, and used proteomics to identify the antibiotic resistance-related protein components of these OMVs. Our results reveal that RC85^+^ OMVs carry proteins that can be involved in degradation of β-lactam antibiotics at higher concentrations than RC85 OMVs. Moreover, RC85^+^ OMVs could hydrolyze β-lactam antibiotics and protect RC85 cells, as well as other species of bacteria, against β-lactam antibiotic-induced killing.

Previous studies showed that OMVs can help bacteria evade the effects of various antibiotics, and that these effects go beyond the transfer of antibiotic resistance genes or the hydrolysis/sequestration of antibiotics and antimicrobial peptides. For example, OMVs were shown to offer immediate protection to bacteria by acting as decoys that can bind or absorb antibiotics^34^. Rumbo *et al*. revealed that OMVs released from carbapenem-resistant *Acinetobacter baumannii* could undertake the horizontal transfer of a carbapenem resistance-related gene to carbapenem-susceptible *A*. *baumannii*^1^. OMVs of *E*. *coli* were shown to sequester the antibiotic, colistin, and degrade the antimicrobial peptide, melittin^7^. Moreover, active OMVs derived from *Moraxella catarrhalis*^12^ and *Staphylococcus aureus*^3^ were found to improve the survival of β-lactam-sensitive bacteria in the presence of β-lactam antibiotics. Consistent with these previous reports, our growth curve experiments and viable bacterial count (Fig. 2) showed that RC85^+^ OMVs could enable β-lactam-susceptible RC85 cells to grow in the presence of β-lactam antibiotics.

To examine whether RC85^+^ OMVs had any specificity or bias in their ability to protect different pathogens, we incubated ampicillin-susceptible *Salmonella* spp. (Sal26B) and *E*. *tarda* (ED45) with RC85^+^ OMVs in the presence of ampicillin (Fig. 3). We found that RC85^+^ OMVs could dose-dependently protect Sal26B and ED45 cells from ampicillin. These results are consistent with previous findings that OMVs from β-lactamase-positive *M*. *catarrhalis* could protect *Streptococcus pneumoniae* and *Haemophilus influenzae* from being killed by amoxicillin, and that OMVs from *E*. *coli* could dose-dependently protect *Pseudomonas aeruginosa* and *Acinetobacter radioresistens* against colistin and melittin^7^. These results suggest that OMVs secreted from antibiotic-resistant bacteria may offer protection to co-existing populations of antibiotic-sensitive bacteria. *E*. *coli* have been associated with intra-abdominal mixed infections involving *Klebsiella pneumoniae*, *Staphylococcus aureus*, *Bacteroides* spp., and *Haemophilus influenzae*^35,36^. If the involved strain of *E*. *coli* has resistance to β-lactam antibiotics, its secreted OMVs could help protect the entire bacterial community related to the mixed infection against β-lactam antibiotics. This could explain why a change of antibiotics (away from β-lactam-based antibiotics) or an increase of antibiotic dosage would be needed to inhibit infection.

Interestingly, our LC-QQQ analysis of the concentration of antibiotics in a cell-free system showed that RC85^+^ OMVs, but not RC85 OMVs, could dose-dependently degrade β-lactam antibiotics (Fig. 4). This could explain why the three strains of antibiotic-susceptible bacteria could resist β-lactam antibiotic-induced killing when treated with RC85^+^ OMVs but not RC85 OMVs, and further suggests that RC85^+^ OMVs possess substances that can degrade β-lactam antibiotics. Indeed, our results (see, for example, Figs 2a, 3a, and Table 1) showed that RC85 and Sal26B cells grown with their MICs of ampicillin (8 μg/mL in both cases) in the presence of 1 μg/mL RC85^+^ OMVs grew at 48 h and 36 h, respectively. This likely reflects that the RC85^+^ OMVs could degrade 20 μg/mL ampicillin to 9.6 μg/mL (which is near the MIC) at 36 h (Fig. 4). In contrast, ED45 cells, for which the MIC of ampicillin is 4 μg/mL, failed to survive in ampicillin-containing medium supplemented with 1 μg/mL RC85^+^ OMVs (Fig. 3a). This suggests that RC85^+^ OMVs cannot degrade 20 μg/mL ampicillin to 4 μg/mL (or thereabouts) over 84 h. These LC-QQQ-based investigations clearly show that OMVs can dose-dependently degrade β-lactam antibiotics and this is the first time to directly show the role of OMVs against ampicillin by using LC-QQQ.

Antibiotic resistance-related proteins were previously detected in some OMVs, hinting at a possible role of these vesicles in antibiotic resistance. For example, Kulkarni *et al*. showed that OMVs of *E*. *coli* possessed proteases and peptidases that may be involved in their ability to sequester colistin and degrade melittin^7^. The presence of active β-lactamase in OMVs from *Moraxella catarrhalis*, and *Staphylococcus aureus* was shown to improve the survival of β-lactam-sensitive bacteria in the presence of β-lactam antibiotics by hydrolyzing β-lactam antibiotics^3,12^. In the present work, our observation that proteinase K blocked the protective effect of RC85^+^ OMVs (Fig. 5d) suggested that these OMVs possess important β-lactam-related proteins, prompting us to compare the protein compositions of RC85^+^ and RC85 OMVs by LC-ESI-MS/MS.

The identified differentially expressed proteins included a number of interesting proteins involved in the mechanism of OMVs to protect bacteria against β-lactam antibiotics (see Dataset 4). Blc1 belongs to a group of ESBLs that exhibit a striking substrate preference for β-lactam antibiotics^24^. RC85^+^ OMVs possessed significantly more (by 4.44-fold) Blc1 than RC85 OMVs (Dataset 4). OmpC and OmpF are porin protein molecules, which form pores in the outer membrane that permit small hydrophobic molecules to diffuse directly into the periplasmic space of *E*. *coli*^28,37^. The action of OmpC porins is involved with permeation of β-lactam compounds through the outer membrane of *E*. *coli*^38,39^. OmpF permits the rapid influx of hydrophilic drugs (e.g., ampicillin, tetracycline, cefoxitin, cephalothin, and doxycycline) into *E*. *coli* cells, explaining why strains with loss of outer membrane porin (OmpF) show decreased susceptibility to such drugs^28^. For OmpW, Tiwari *et al*. proposed that this protein was involved in the uptake of β-lactam antibiotics by *Acinetobacter baumannii*, and showed that decreased OmpW reduced the entrance of β-lactam antibiotics into cells, barring the antibiotics from reaching their target proteins^40^. TolC is affiliated with the multidrug efflux pump system, which functions when bacteria are exposed to antibiotics such as ampicillin, tetracycline, and kanamycin^27,31^.

The four major mechanisms by which bacteria can resist β-lactam antibiotics are as follows: 1) Production of β-lactamase enzymes; 2) Changes in the active site of PBPs (penicillin binding proteins); 3) Decreased expression of outer membrane proteins; and 4) Overproduction of efflux pump^41^. The β-lactam antibiotic must pass through the traverse porin channel in the outer membrane to access the PBP of the inner plasma membrane. However, proteins produced by mutations in porin-encoding genes or porin loss show lower permeability to β-lactam antibiotics^42,43^. Efflux pumps can export a wide range of substrates from the periplasm to the surrounding environment^44^, thus overproduction of efflux pump can augment the resistance of β-lactam antibiotics^45^.

In the present study, we found that Blc1, OmpC, OmpF and OmpW were upregulated and TolC was downregulated in RC85^+^ OMVs versus RC85 OMVs (Dataset 4). Not only the amount of β-lactamase but also the transfer of β-lactam antibiotics to the enzyme is important in determining the resistance spectrum^46^. The concentration of β-lactam antibiotics in periplasm depends on the function of porin and efflux pumps on the outer membrane. The increase in porin entry channels and the decrease in efflux pump may increase the concentration of β-lactam substrate in OMVs and further augment the β-lactamase enzyme efficiency. Thus, we speculate that secreted RC85^+^ OMVs might take up β-lactam antibiotics into their lumens through increased quantity of OmpC, OmpF and OmpW, preventing a portion of the molecules from reaching the bacterial cell, whereas the β-lactam antibiotics that enter the RC85^+^ OMVs might rarely be effluxed because of decreased quantity of TolC. Therefore, increased quantity of β-lactamase enzyme (Blc1) might have augmented the efficiency for hydrolyzing β-lactam antibiotics confined in lumens of OMVs. For these five proteins (Dataset 4) were packaged into OMVs and secreted to obstruct antibiotics extracellularly by uptake, degradation, and efflux and such approach will be helpful for further understanding the novel mechanisms of OMVs against β-lactam antibiotic resistance. Our lab is currently working to characterize how loss of each proteins related to the mechanism of OMVs against β-lactam antibiotics by using gene deletion method to develop a more complete understanding of how these proteins play specific roles in protecting bacteria against β-lactam antibiotics.

Furthermore, a number of proteins related to other antibiotics or antimicrobial peptides were found in both OMVs (see Dataset 5). Lin *et al*. showed that downregulation of LamB was reported to increase the resistance of *E*. *coli* cells against chlortetracycline^47^. Several studies revealed that quantity alteration of LamB could develop the resistance of *E*. *coli* against ampicillin, streptomycin, and tetracycline^26,27,48^. Lloblet *et al*. suggested that the absence of OmpA was involved in susceptibility to antimicrobial peptides, such as protamine, human neutrophil α-defensin 1, and polymyxin B^32^. Protease 7 (omptin, OmpT) has been shown to degrade the antimicrobial peptide, protamine^7,33^ and the antibiotic, colistin^21^. With respect to MipA, Zhang *et al*. showed that gene deletion of mipA increased the MIC of kanamycin in kanamycin-resistant *E*. *coli*^31^. Altered FadL protein has been shown to promote the resistance of *E*. *coli* to streptomycin^49^ and decrease MIC of *E*. *coli* to chlortetracycline^47^. Tsx is a nucleoside-specific channel-forming integral Omp that functions as a substrate-specific transporter of nucleosides, and it was shown to be up-regulated in cells subjected to ten sequential subcultures in tetracycline, compared to the ancestor strain^27^. Our observation that the above proteins were present in both RC85^+^ OMVs and RC85 OMVs suggested that OMVs contain numerous proteins related to various antibiotics and antimicrobial peptides. These proteins may be important for the survival of bacteria, and our findings suggest that the protective role of OMVs may be not limited to β-lactam antibiotics, but could extend to other antibiotics.

The proteins of OMVs have been suggested to arise from various origins, including the cytoplasmic, inner membrane, outer membrane, periplasmic, and extracellular compartments, but the entrapment mechanisms of these proteins were previously unknown^50,51^. These findings were consistent between our GO analysis (Fig. 6c,f; see Dataset 3) and SDS-PAGE assay (Fig. 1g,h), which indicated that the RC85^+^ and RC85 OMVs include proteins of various subcellular origins. OMVs with membranes whose surface compositions are nearly identical to the bacterial outer membrane^52^ could provide a target for antibiotics that target the bacterial membrane^53^. Thus, released OMVs might help defend cells from antibiotics by acting as decoys^34^ that bind antibiotic molecules before they reach the main body of the bacterium. Our predictions of the biological processes (Fig. 6b,e) and molecular functions (Fig. 6d,g) of the OMV proteins thus improve our understanding of the pathophysiological functions, biogenesis, and potential action mechanisms of OMVs.

Since β-lactamase was identified in our LC-ESI-MS/MS analysis of OMV proteins, we tested whether this protein was involved in the protective effects of OMVs, as assessed by β-lactamase activity assays (Fig. 7a,b) and growth curve experiments in the presence of a β-lactamase inhibitor (Fig. 7c). Our β-lactamase activity assay indicated that RC85^+^ OMVs had about 3.1-fold higher β-lactamase activity compared to RC85 OMVs (Fig. 7b). Moreover, RC85^+^ OMVs treated with a β-lactamase inhibitor failed to protect RC85 cells against ampicillin, cefoperazone, and cefotaxime (Fig. 7c). Hence, the ability of RC85^+^ OMVs to degrade β-lactam antibiotics (Fig. 4) appears to rely largely on the hydrolytic ability of β-lactamase, which destroys the stability of the β-lactam ring to destroy the antibacterial properties of such agents. Since β-lactamase is located in the bacterial periplasm and periplasmic and inner membrane proteins can be encapsulated during OMV formation^12^, our results suggest that RC85^+^ OMVs can harbor β-lactamase to function as a first line of bacterial defense against β-lactam antibiotics. Clavulanic acid and sulbactam can penetrate the outer membrane^54^, and thus likely entered the OMVs to inhibit the action of their β-lactamase. As OMVs are freely permeable to a number of antibiotics^29^, we propose that the tested β-lactam antibiotics (ampicillin, cefoperazone, and cefotaxime) penetrate the outer membrane of OMVs, enter the lumen where the β-lactamase is located, and become degraded/inactivated. This may be a novel mechanism of bacterial protection and antibiotic resistance.

To summarize, we herein show that: 1) OMVs naturally secreted from β-lactam antibiotics-resistant *E*. *coli* harbor a number of antibiotic-related proteins originating from diverse subcellular locations; and 2) these OMVs contribute to the defense and survival of β-lactam-susceptible *E*. *coli* and other species of bacteria by degrading β-lactam antibiotics. It is crucial that we elucidate all possible mechanisms of bacterial resistance, which is currently causing a global problem. OMVs are the interesting novel carriers of antibiotic related proteins, and we herein show that these vesicles are essential for the growth and survival of bacteria in the presence of antibiotics. Therefore, our findings on how bacteria utilize OMVs may consequently pave the way to overcome multi-drug resistant bacteria.

## Methods

### Bacterial strains

Antimicrobial-sensitive *Escherichia coli* RC85^55^ and multidrug-resistant *Salmonella* sp., Sal45^56^ were used as the recipient and donor strains, respectively, in a filter-mating conjugation assay to produce the antimicrobial-resistant strain, RC85^+^^57^. Tryptone soya broth (TSB; Oxoid, United Kingdom) or tryptone soya agar (TSA; Oxoid) were used to grow RC85 and RC85^+^ cells at 37 °C. The ampicillin-susceptible bacteria, *Salmonella* spp. Sal26B^56^ and *Edwardsiella tarda* ED45^20^, were cultured on brain heart infusion (BHI; Oxoid) agar and TSA, respectively, at 37 °C.

### Determination of minimum inhibitory concentrations

Three antimicrobial agents known to confer bactericidal effects by inhibiting cell wall biosynthesis, namely ampicillin, cefotaxime, and cefoperazone (Sigma-Aldrich, USA) were selected. The minimum inhibitory concentration (MIC) of each antimicrobial agent was investigated in RC85 and RC85^+^ cells by the broth-dilution method using 96-well plates^58^. Detailed information about the methods of determination of minimum inhibitory concentration can be found in the Supplementary Information.

### Isolation of pure OMVs

OMVs were isolated and purified from cultures of RC85^+^ and RC85 by multiple filtration, QuixStand Benchtop system (GE Healthcare, Sweden), and ultracentrifugation followed by visualization using transmission electron microscopy (TEM) and dynamic light scattering (DLS). Detailed information about the methods of OMVs purification, visualization by TEM, and particle size distribution by DLS can be found in the Supplementary Information.

### Subcellular fractionation

Whole cell lysates (WCLs), periplasmic proteins (PPs), cytoplasmic proteins (CPs), outer membrane proteins (OMPs), and inner membrane proteins (IMPs) from RC85^+^ and RC85 cells were purified as described previously with some modifications^20,21,59^. Detailed information about the methods of subcellular fractionation can be found in the Supplementary Information.

### Electrophoresis and in-gel digestion

Three micrograms of WCLs, PPs, CPs, IMPs, OMPs and OMVs from RC85^+^ and RC85 cells were mixed with sample buffer and were separated by SDS-PAGE, according to the method of Laemmli^60^. The resolved proteins were visualized by silver staining^61^. For in-gel digestion, which was performed as previously described^20^, 60 μg of OMVs from RC85^+^ and RC85 cells were resolved on a separating gel. Each gel lane was cut into six slices, and each slice was destained, dehydrated, dried, and subjected to in-gel digestion. The tryptic peptides were extracted into 10% (v/v) formic acid and acetonitrile, followed by drying in a vacuum centrifuge prior to LC-MS/MS analysis. Detailed information about the methods of electrophoresis and in-gel digestion can be found in the Supplementary Information.

### LC-MS/MS

LC-MS/MS analysis was carried out as previously described^20,62^, with some modifications. Each obtained peptide mixture was resuspended in 0.1% TFA and injected into an analytical column (Zorbax 300SB-C18 75 um i.d. × 15 cm column; Agilent, Germany) via a trap column (Zorbax 300SB-C18 300 μm i.d. × 5 mm column; Agilent). The peptides were separated in an acetonitrile gradient of buffer A (0.1% formic acid in water) and buffer B (0.1% formic acid in pure acetonitrile) at a constant flow rate of 0.2 μl/min, using an Agilent 100 series nano HPLC system coupled on-line to a LTQ ion-trap mass spectrometer (Thermo Fisher Scientific). The gradient commenced with 5% B, rose linearly to 40% B over 100 min, increased to 80% B over 1 min, and then isocratically increased to 80% B over 15 min. A full-scan mode (*m/z* 350–1600) was enabled, and each survey MS scan was followed by three MS/MS scans using the 30-sec dynamic exclusion option set. The utilized ESI-Q-TOF ion source parameters were as follows: ion spray voltage, 2.2 kV; capillary voltage, 24 V; and capillary temperature, 200 °C. The rolling collision energy was set to 35%.

### Quantitative protein profiling, statistics and database searching

The obtained LC/MS data were analyzed with the DeCyder MS software (version 2.0; GE Healthcare, Uppsala, Sweden). The MS/MS spectra of the peptide peaks were searched against the SwissProt bacterial database using Mascot^TM^ 2.3 (Matrix Science, London, UK). Detailed information about the methods of quantitative protein profiling, statistics and database searching can be found in the Supplementary Information.

### *In silico* analysis of functional associations

Software Tool for Researching Annotations of Proteins (STRAP) version 1.5 (Boston University School of Medicine, USA) was used to classify the obtained differentially expressed proteins by their gene ontology (GO) terms, such as biological process, cellular component, and molecular function.

### Effect of OMVs on the growth of bacteria in the presence of antibiotics

The effects of RC85^+^ and RC85 OMVs against the cytotoxicity of β-lactam antibiotics were monitored by assessing the growth curves of OMV-treated RC85 cells. The following antibiotics were used at the following concentrations: ampicillin, 30 μg/mL; cefoperazone, 4 μg/mL; and cefotaxime, 1.25 μg/mL. Cultured RC85 cells were separately inoculated into media containing each antibiotic and various concentrations of OMVs from RC85^+^ or RC85 cells. Cultured Sal26B and ED45 cells were inoculated to medium containing 30 μg/mL ampicillin plus RC85^+^ or RC85 OMVs. The bacterial growth curves at OD_600_ were recorded at 12-h intervals for 84 h. Experiments were performed using bacterial cultures from three independent batches. After 84 h, the bacterial cultures were streaked on TSA with and without the respective antibiotics to test whether the OMVs could confer resistance to antibiotic-susceptible bacteria. Detailed information about the methods of effect of OMVs on the growth of bacteria in the presence of antibiotics can be found in the Supplementary Information.

### Efficacy of OMVs in restoring growth in the presence of antibiotics

To evaluate the ability of RC85^+^ and RC85 OMVs to protect RC85 cells against growth-inhibiting concentrations of antibiotics, we carried out quantitative plate assays as previously described^21^, with some modifications. Colonies from each cultured sample (*n* = 5, colonies per sample) were randomly chosen and identified by matrix-assisted laser desorption ionization-time of flight mass spectrometry (MALDI-TOF MS), performed as previously described^63^, to test for contamination. Detailed information about the methods of efficacy of OMVs in restoring growth in the presence of antibiotics can be found in the Supplementary Information.

### Effect of OMVs treated with proteinase K, DNase I, or RNase A

To determine which substances of OMVs play key roles in their protective effects against antibiotics, we measured the growth curves of RC85 cells in the presence of 30 μg/mL ampicillin and 30 μg RC85^+^ OMVs that had been pre-treated with proteinase K, DNase I, or RNase A. Cells were also treated with plasmid DNA (pDNA) or genomic DNA (gDNA) extracted from RC85^+^ instead of RC85^+^ OMVs under the same experimental conditions, to test whether antibiotic-resistance genes from RC85^+^ can affect to survival of RC85 cells. In all cases, growth curves at OD_600_ were recorded every 12 h from 0 h to 84 h. All experiments were separately conducted in triplicate. Detailed information about the methods of effect of OMVs treated with proteinase K, DNase I, or RNase A can be found in the Supplementary Information.

### Measurement of antibiotic concentrations

To evaluate whether OMVs could directly degrade antibiotics, the effects of RC85^+^ and RC85 OMVs on the concentrations of three antibiotics in a cell-free system were performed by liquid chromatography/electrospray ionization mass spectrometry (LC-ESI-QQQ-MS/MS; 6420 Triple Quad LC/MS; Agilent). Supplementary Table S4 shows the optimized conditions for each antibiotic. The MassHunter software (version B.06.00; Agilent) was used to process the LC-MS/MS data and quantify the analytes. Detailed information about the methods of measurement of antibiotic concentrations can be found in the Supplementary Information.

### Quantification of β-lactamase activity

To test for differences in β-lactamase activity between cell extracts of RC85^+^ and RC85 cells, supernatants from RC85^+^ and RC85 cell cultures, and OMVs from RC85^+^ and RC85 cells, we used a colorimetric β-lactamase activity assay kit (BioVision, Canada) according to the manufacturer’s instructions. The assay involves the hydrolysis of nitrocefin, a chromogenic cephalosporin, which produces a colored product that is measured by spectrophotometry (OD_490_) in kinetic mode for 60 min at 25 °C. The quantity of enzyme capable of hydrolyzing 1.0 μM of nitrocefin per minute at 25 °C corresponds to 1 U of β-lactamase^64^. Detailed information about the methods of quantification of β-lactamase activity can be found in the Supplementary Information.

### β-Lactamase inhibitors

The effects of the β-lactamase inhibitors, clavulanic acid (Wako Pure Chemical Industries, Ltd., Japan) and sulbactam (Abcam, United Kingdom), were investigated by growth curve experiments performed as described above. The β-lactamase inhibitors were added at the previously described fixed concentrations^65^ with some modification, for final concentrations of 25 μg/mL in each case. Briefly, the growth curves of RC85 cells treated with antibiotics (ampicillin, 30 μg/mL; cefoperazone, 4 μg/mL; cefotaxime, 1.25 μg/mL) plus clavulanic acid or sulbactam were determined in the presence of 50 μg/mL RC85^+^ OMVs. The bacterial cultures were incubated at 37 °C with gentle shaking and the OD_600_ was recorded every 12 h for 84 h from three independent batches.

### Statistical analysis

Statistical calculations were done using IBM SPSS Statistics software, version 19 (SPSS, Inc., USA). One-way Analysis of Variance (ANOVA) and Tukey’s multiple comparison test were used to compare the sample groups (GraphPad Prism version 7.0.1; GraphPad, CA). All data are expressed as means ± standard errors of the means (SEMs). Differences were considered statistically significant at *P* < 0.05.

### Data availability

All data generated or analyzed during this study are included in this published article and its Supplementary Information files.

## Electronic supplementary material

Supplementary information

Dataset 1

Dataset 2

Dataset 3

Dataset 4

Dataset 5
