# Supplementary material for: Outer membrane vesicles from β-lactam-resistant Escherichia coli enable the survival of β-lactam-susceptible E. coli in the presence of β-lactam antibiotics
Source: Sci Rep. 2018 Mar 29;8:5402. doi: 10.1038/s41598-018-23656-0 (PMC5876404; doi:10.1038/s41598-018-23656-0)
Supplement: Supplementary file 1 — Supplementary information [file 41598_2018_23656_MOESM1_ESM.docx]

**Outer membrane vesicles from β-lactam-resistant *Escherichia coli* enable the survival of β-lactam-susceptible *E. coli* in the presence of β-lactam antibiotics**

Si Won Kim^1,+^, Seong Bin Park^2,+^, Se Pyeong Im^1^, Jung Seok Lee^1^, Jae Wook Jung^1^, Tae Won Gong^1^, Jassy Mary S. Lazarte^1^, Jaesung Kim^1^, Jong-Su Seo^3^, Jong-Hwan Kim^3^, Jong-Wook Song^3^, Hyun Suk Jung^4^, Gwang Joong Kim^4^, Young Ju Lee^5^, Suk-Kyung Lim^6^, and Tae Sung Jung^1,*^

^1^Laboratory of Aquatic Animal Diseases, Institute of Animal Medicine, College of Veterinary Medicine, Gyeongsang National University, Jinju, 52828, Republic of Korea ^2^Department of Animal and Dairy Sciences, Mississippi State University, MS, 39762, USA

^3^Environmental Chemistry Research Center, Korea Institute of Toxicology Gyeongnam Department of Environmental Toxicology and Chemistry, Jinju, 52834, Republic of Korea

^4^Department of Biochemistry, College of Natural Sciences, Kangwon National University, Chuncheon, 24341, Republic of Korea

^5^College of Veterinary Medicine, Kyoungpook National University, Daegu, 41566, Republic of Korea

^6^Bacterial Disease Division, Animal and Plant Quarantine Agency, Gimcheon-si, 39660, Republic of Korea

^*^Address correspondence to Tae Sung Jung, jungts@gnu.ac.kr

^+^these authors contributed equally to this work.

**SUPPLEMENTARY METHODS**

**Determination of minimum inhibitory concentrations (MIC) values**

RC85 and RC85^+^ cells were grown in Luria-Bertani (LB; Oxoid) broth, and 5 × 10^5^ colony forming unit (CFU)/mL was inoculated to a 96-well plate loaded with LB containing two-fold serial dilutions of the abovementioned antibiotics ranging from 0.25 µg/mL to 256 µg/mL. The same procedure was used to determine the MICs of ampicillin against Sal26B and ED45. The cells were incubated at 37°C for 24 h, and the optical density at 600 nm (OD_600_) was measured by spectrophotometry. The lowest antibiotic concentration in which a given bacterium without increase of OD was regarded as the MIC. The presented MIC values were presented as mean ± SEM using three independent experiments.

**OMVs purification**

Single colonies of RC85^+^ and RC85 were separately inoculated to 1.7 ml of LB broth and incubated at 37°C for 12 h with shaking at 150 rpm. The cultures were re-inoculated to 5.5 L of TSB and incubated at 37°C for 4 h 30 min (RC85^+^) or 6 h (RC85) with shaking at 150 rpm. Each culture was centrifuged at 6,000 × *g* for 20 min, and the supernatant was subjected to filtration using 0.45-μm pore-sized vacuum filters. Each filtered supernatant was concentrated about 33-fold by ultrafiltration using a 100-kDa hollow fiber membrane and the QuixStand Benchtop system (GE Healthcare, Sweden), and further purified using a 0.2-μm syringe filter (Thermo Fisher Scientific, IL). Each supernatant was centrifuged at 150,000 × *g* at 4°C for 3 h, and the crude OMV pellet was washed and resuspended in 10 mM Tris-HCl (pH 8.0) (Biopure, Korea). Further purification was performed on a continuous sucrose density gradient at 120,000 × *g* at 4°C for 18 h. The OMV band was removed and filtered through a 0.2-μm filter with about 20 mL of 10 mM Tris-HCl pH 8.0. This sample was centrifuged for 3 h at 150,000 × *g* at 4°C. The final OMV pellet was washed and resuspended in 10 mM Tris-HCl (pH 8.0) and filtered through a 0.2-μm filter. The resulting OMV suspensions were checked on LB agar to confirm that the preparations were free of bacteria. All purification steps were performed at 4°C. The protein yields of OMV samples from RC85^+^ and RC85 cells were measured using a Pierce BCA protein assay kit (Thermo Fisher Scientific).

**Visualization of OMVs**

OMVs isolated from RC85^+^ and RC85 cells were placed on a carbon-coated grid (Ted Pella, USA) that had been glow-discharged for 3 min in air. The grid was immediately subjected to negative staining with 1% (w/v) uranyl acetate, and then examined using a Tecnai G2 Spirit Twin TEM system (FEI, USA) under an acceleration voltage of 120 kV.

**Particle size distribution and measurement of zeta potential**

Each OMV was characterized by measurements of the particle size distribution and zeta potential. The diameters of the OMV preparations obtained from RC85 and RC85^+^ cells were determined at 25°C by dynamic light scattering (DLS) using a Nano ZS instrument (Malvern Instruments, Malvern, UK) and the Zetasizer software (version 7.11; Malvern Instruments). Three independent measurements (15 experimental runs for each measurement) were averaged to obtain an average intensity-weighted diameter. All data met the quality criteria (polydispersity index lower than 0.3). The zeta potential was measured using a Nano ZS instrument (Malvern) with a Helium-Neon laser (633 nm) at 25°C. Each experiment was carried out in triplicate.

**Subcellular fractionation**

For preparation of WCLs, RC85^+^ and RC85 cells were grown in LB for 12 h at 37°C, pelleted at 5,000 × *g* for 30 min, and washed with PBS. Each cell pellet was suspended to 4 ml/g cells in chilled lysis buffer (50 mM Tris-HCl, pH 7.5, 100 mM NaCl, 5 mM DTT, and 1 mM PMSF), incubated for 10 min, and sonicated for ten cycles of 10 sec bursts with 30 sec cooling intervals. The resulting crude extracts were centrifuged at 12,000 × *g* for 5 min at 4°C for removal of cell debris. Each WCL pellet was resuspended in 10 mM Tris-HCl (pH 8.0). For preparation of the various fractions, pelleted cells were suspended at 4 ml/g cells in 20% (w/v) sucrose dissolved in 20 mM Tris-HCl (pH 8.0) containing 0.1 M EDTA (0.2 ml/g cells) and lysozyme (600 µg/g cells). The mixtures were incubated on ice for 40 min, 0.5 M MgCl_2_ (0.16 ml/g cells) was added, and the ice incubation was continued for an additional 10 min. Spheroplasts were removed by centrifugation at 9,500 × *g* for 20 min at 4°C, and the PP-containing supernatant was stored at -80°C until use. For preparation of membrane proteins (MPs), the above-obtained spheroplasts were resuspended in ice-cold 10 mM Tris-HCl (pH 8.0) and sonicated on ice by 12 cycles of 30 sec bursts with 30 sec cooling intervals. The sample was centrifuged at 8,000 × *g* for 5 min to remove cell debris, and the supernatant was centrifuged at 40,000 × *g* for 1 h to pellet the cell membrane material. The supernatant was collected as the CP fraction and stored at -80°C. The membrane pellet was washed in 10 mM Tris-HCl (pH 8.0), resuspended in ddH_2_O, freeze-thawed, and incubated in 2% (w/v) sarkosyl (sodium N-lauroylsarcosinate) at 25°C for 20 min. The mixture was centrifuged at 40,000 × *g* for 90 min at 4°C, the pelleted OMPs were resuspended in 10 mM Tris-HCl (pH 8.0), and the IMP-containing supernatant was stored at -80°C until use. The protein concentrations of the WCLs, PPs, CPs, OMPs, and IMPs were quantified using a BCA protein assay kit.

**Electrophoresis and in-gel digestion**

Sodium dodecyl sulfate-polyacrylamide gel electrophoresis (SDS-PAGE) was performed using a 12% (w/v) acrylamide separating gel. Briefly, equal amounts of WCLs, PPs, CPs, IMPs, OMPs and OMVs were mixed with 5 × sample buffer (5:1 v/w ratio of buffer to sample) containing 60 mM Tris-HCl, 25% (v/v) glycerol, 2% (w/v) SDS, 14.4 mM β-mercaptoethanol, and 0.1% (w/v) bromophenol blue. The samples were boiled for 10 min, cooled on ice for 10 min, and 3 μg of each sample was subjected to SDS-PAGE until the dye-front reached the bottom of the gel. The resolved proteins were visualized by silver staining. For in-gel digestion, 60 μg of OMVs from RC85^+^ and RC85 cells were resolved on a 12% (w/v) separating gel and stained with Bio-safe Coomassie G-250 (Bio-Rad, Hercules, CA). Each gel lane was cut into six slices, and each slice was destained with 500 μl of 40% (v/v) ethanol in 75 mM ammonium bicarbonate (ABC), treated with DTT (0.0509 g iodoacetamide in 5 ml of 25 mM ABC), dehydrated with 300 μl acetonitrile (ACN) for 30 min at 37°C, dried, and subjected to in-gel digestion with 20 ng/ml of sequencing-grade modified trypsin (Promega, Madison, WI) at 37°C overnight. The tryptic peptides were extracted into 30 μl 10 % (v/v) formic acid and acetonitrile, followed by drying in a vacuum centrifuge.

**Quantitative protein profiling, statistics and database searching**

Peptide peaks were detected with an average peak width of 1 min and matched with a mass accuracy of at least 0.6 Da. The processed data were manually inspected and overlapping peaks were discarded. A differentially expressed protein was defined as showing a two-fold or greater increase/decrease in comparable intensity or the complete appearance/disappearance of a spot. After alignment of the retention times of the chromatogram, normalization was performed with the measured intensity distribution and the proteome was quantified with the peak intensity ratio. The MS/MS spectra of the peptide peaks were searched against the SwissProt bacterial database using Mascot^TM^ 2.3 (Matrix Science, London, UK). The search parameters were set as follows: enzyme, trypsin; missed cleavage sites allowed, 2; precursor mass tolerance, 2 Da; and fragment mass tolerance, 1 Da. During analysis, carbamidomethyl (C) modification was set as fixed, whereas oxidation modification (M) was not. The peptide identifications were imported to DeCyder MS software. For quantitative profiling, we selected proteins identified by multiple peptides having significant Mascot scores (*p*<0.05).

**Effect of OMVs on the growth of bacteria in the presence of antibiotics**

The growth-inhibiting concentration of each antibiotic against RC85 cells was determined by the broth dilution method. Cultured RC85 cells were diluted, 5 × 10^5^ CFU/mL was separately inoculated into media containing each antibiotic (ampicillin, 30 μg/mL; cefoperazone, 4 μg/mL; and cefotaxime, 1.25 μg/mL) plus 1, 5, 25, 50 μg/mL OMVs from RC85^+^ cells or 50 μg/mL OMVs from RC85 cells. To test whether OMVs from *E. coli* can influence different genera of bacteria, Sal26B and ED45 cells were cultured in BHI and TSB, respectively, at 37°C, and 5 × 10^5^ CFU/mL was inoculated to medium containing 30 μg/mL ampicillin plus 1, 5, 25, 50 μg/mL RC85^+^ OMVs or 50 μg/mL RC85 OMVs. The positive control consisted of bacteria in antibiotic-free medium, and the negative control contained bacteria plus growth-inhibiting concentrations of the respective antibiotics. All tubes were incubated at 37°C with shaking at 150 rpm. All experiments were conducted in the dark to exclude the influence of light on the stability of the utilized antibiotics. The bacterial growth curves at OD_600_ were recorded at 12-h intervals for 84 h using an xMark microplate spectrophotometer (Bio-Rad).

**Efficacy of OMVs in restoring growth in the presence of antibiotics**

The samples collected at 12-h intervals for 84 h in the growth curve experiment (described above) were serially diluted by ten-fold in PBS (from 10^-1^ to 10^-6^) and 20 μl of each sample was spread on TSA. The plates were incubated at 37°C for 14 to 16 h, and colonies were counted. The count obtained in the absence of antibiotics was taken as 100%, and the corresponding counts in the presence of different concentrations of RC85^+^ or RC85 OMVs plus the respective antibiotics were calculated. Three independent trials were performed.

**Effect of OMVs treated with proteinase K, DNase I, or RNase A**

The pDNA and gDNA were extracted from RC85^+^ cells using a plasmid DNA purification kit (iNtRON, South Korea) and a genomic DNA prep kit (SolGent, South Korea), respectively, and the purity and quantity of DNA were determined using a NanoDrop 1000 spectrophotometer (Thermo Fisher Scientific). For enzyme experiments, RC85 cells were cultured in LB broth, and then 5 × 10^5^ CFU/mL was mixed in LB containing 30 μg/mL ampicillin and 30 μg purified RC85^+^ OMVs pre-treated with the following: 50 or 100 μg/mL proteinase K (Invitrogen, CA) for 1 h at 37°C; 2.5 or 5 U of DNase I (Invitrogen) for 15 min at room temperature and addition of 25 mM EDTA solution to the reaction mixture to inactivate DNase I; 100 or 200 μg/mL RNase A (iNtRON) for 30 min at 37°C and addition of RNase OUT (Invitrogen) to the reaction mixture for 30min at 37°C; or 50 or 100 μg/mL proteinase K plus a protease inhibitor cocktail (Sigma-Aldrich) for 1 h at 37°C. For pDNA and gDNA experiments, cultured RC85 cells (5 × 10^5^ CFU/mL) were directly treated with 2.5 μg of the isolated pDNA or gDNA plus 30 μg/mL ampicillin. The bacterial cultures were incubated at 37°C with constant shaking (150 rpm) and bacterial growth curves at OD_600_ were recorded every 12 h from 0 h to 84 h, using a microplate reader.

**Measurement of antibiotic concentrations**

One microgram per milliliter or 10 μg/mL of RC85^+^ OMVs or 10 μg/mL of RC85 OMVs in PBS were mixed with ampicillin (20 μg/mL), cefoperazone (5 μg/mL), or cefotaxime (5 μg/mL). Filtered PBS containing the respective antibiotics without OMVs were used as a positive control. All samples were incubated at 37°C with shaking at 150 rpm, and diluted 20-fold for measurements. The concentrations of antibiotics were recorded at 18-h intervals for 36 h in triplicate. Supplementary Table S3 shows the optimized conditions for each antibiotic. For LC-MS/MS, LC-MS grade water (Burdick & Jackson, USA) containing 5 mM ammonium formate (Sigma-Aldrich) and 0.1% formic acid (KANTO, Japan) (v/v) (solution A) and LC grade methanol (Burdick & Jackson) containing 5 mM ammonium formate with 0.1% formic acid (v/v) (solution B) were used as the mobile phase, at an initial A:B ratio of 30:70 or 50:50, depending on the antibiotic in question (Table S3). The compounds were separated using a Poroshell 120 EC-C18 column (2.1 × 100 mm, 2.7 μm; Agilent). Isocratic elution with phases A and B was followed by 3 min of total chromatography. The flow rate was 0.2 mL/ min, the injection volume was set to 5 μl (ampicillin) or 1 μl (cefoperazone, cefotaxime), the column temperature was 30ºC, and 99.99% pure nitrogen gas was used for desolvation. For quantification of antibiotics, we selected at least two transitions for each analyte and used the positive electric spray ionization (ESI+) with multiple reaction monitoring (MRM) mode. The MassHunter software (version B.06.00; Agilent) was used to process the LC-MS/MS data and quantify the analytes.

**Quantification of β-lactamase activity**

RC85^+^ and RC85 cultures were grown in LB broth at 37ºC for 12 h and centrifuged at 10,000 × *g* for 10 min. Each OMV-containing supernatant was passed through a 0.2-μm syringe filter and stored at 4ºC. The obtained pellets were sonicated for 5 min, cooled on ice for 5 min, and centrifuged at 16,000 × *g* at 4ºC for 20 min. A BCA protein assay kit was used to determine the protein concentrations of the crude cell extracts prepared by sonication, the culture supernatants obtained from fresh cultures of RC85^+^ and RC85 cells, and the OMVs prepared from RC85^+^ and RC85 cells. Equivalent concentrations (10 μg) of each sample were dispensed to the wells of a clear flat-bottomed 96-well, and the provided nitrocefin and buffer were added to a final volume of 100 μl. The OD_490_ was immediately measured in kinetic mode. For all measurements, three independent experiments were performed. A standard curve was generated using 0, 4, 8, 12, 16, and 20 nM of nitrocefin, and the specific β-lactamase activity of each sample was expressed in milliunits per milligram of protein.

**SUPPLEMENTARY TABLES**

**Table S1. Growth inhibition zone diameters of several antimicrobial agents against bacterial strains using disc diffusion method.**

| Antimicrobial agent | Disk content | Growth inhibition zone (mm)^a^ | | |
| --- | --- | --- | --- | --- |
|  |  | Sal45 | RC85 | RC85^+^ |
| Ampicillin | 10 μg | 0 | 13 | 0 |
| Amikacin | 30 μg | 16 | 16 | 16 |
| Amoxicillin-clavulanate (2:1) | 20/10 μg | 10 | 16 | 10 |
| Cefazolin | 30 μg | 0 | 20 | 0 |
| Cefepime | 30 μg | 0 | 24 | 0 |
| Cefotaxime | 30 μg | 0 | 25 | 0 |
| Cefoxitin | 30 μg | 22 | 25 | 22 |
| Cephalothin | 30 μg | 0 | 13 | 0 |
| Chloramphenicol | 30 μg | 25 | 25 | 25 |
| Ciprofloxacin | 5 μg | 21 | 21 | 21 |
| Clindamycin | 2 μg | 0 | 0 | 0 |
| Enrofloxacin | 5 μg | 19 | 19 | 19 |
| Erythromycin | 15 μg | 0 | 0 | 0 |
| Gentamicin | 10 μg | 0 | 18 | 0 |
| Imipenem | 10 μg | 22 | 24 | 23 |
| Norfloxacin | 10 μg | 14 | 18 | 15 |
| Oxacillin | 1 μg | 0 | 0 | 0 |
| Penicillin | 10 units | 0 | 0 | 0 |
| Rifampin | 5 μg | 0 | 0 | 0 |
| Streptomycin | 10 μg | 0 | 12 | 0 |
| Tetracycline | 30 μg | 0 | 20 | 0 |
| Trimethoprim-sulfamethoxazole (1:19) | 1.25/23.75 μg | 21 | 16 | 23 |
| ^a^Data are the averages of duplicate experiments. | |  |  |  |

**Table S2. Physical characterization of OMVs secreted from RC85^+^ and RC85.**

|  | Size (nm) | Polydispersity | Z-potential (mV) |
| --- | --- | --- | --- |
| RC85+ OMV | 86.19±0.49 | 0.269±0.007 | –26.87±1.24 |
| RC85 OMV | 66.90±0.71 | 0.266±0.006 | –26.77±1.68 |

**Table S3. One way ANOVA Multiple comparison statistics with Tukey's multiple comparison test for β-lactamase activity.**

| **Tukey's multiple comparisons test** | **Mean Diff.** | **95.00% CI of diff.** | **Significant?** | **Summary** | **Adjusted P Value** |
| --- | --- | --- | --- | --- | --- |
| RC85+OMV vs. RC85 OMV | 33.87 | 30.3 to 37.44 | Yes | **** | <0.0001 |
| RC85+OMV vs. RC85+ extract | 2.475 | -5.722 to 10.67 | No | ns | 0.3988 |
| RC85+OMV vs. RC85 extract | 38.85 | 34.24 to 43.47 | Yes | **** | <0.0001 |
| RC85+OMV vs. RC85+ supernatant | 33.16 | 27 to 39.32 | Yes | *** | 0.0002 |
| RC85+OMV vs. RC85 supernatant | 39.44 | 34.68 to 44.2 | Yes | **** | <0.0001 |
| RC85 OMV vs. RC85+ extract | -31.39 | -36.12 to -26.67 | Yes | **** | <0.0001 |
| RC85 OMV vs. RC85 extract | 4.984 | 3.471 to 6.497 | Yes | ** | 0.0047 |
| RC85 OMV vs. RC85+ supernatant | -0.7096 | -3.46 to 2.041 | No | ns | 0.4893 |
| RC85 OMV vs. RC85 supernatant | 5.573 | 2.833 to 8.313 | Yes | * | 0.0128 |
| RC85+ extract vs. RC85 extract | 36.38 | 32.76 to 40 | Yes | **** | <0.0001 |
| RC85+ extract vs. RC85+ supernatant | 30.69 | 28.65 to 32.72 | Yes | **** | <0.0001 |
| RC85+ extract vs. RC85 supernatant | 36.97 | 32.63 to 41.3 | Yes | **** | <0.0001 |
| RC85 extract vs. RC85+ supernatant | -5.694 | -7.293 to -4.095 | Yes | ** | 0.0035 |
| RC85 extract vs. RC85 supernatant | 0.5884 | -0.991 to 2.168 | No | ns | 0.2928 |
| RC85+ supernatant vs. RC85 supernatant | 6.283 | 3.662 to 8.903 | Yes | ** | 0.0098 |
| **P* < 0.05, ***P* < 0.01, ****P* < 0.001, *****P* < 0.0001 | | | | | |

**Table S4. Triple quad LC/MS conditions for all analytes.**

|  |  |  |  |  |  |  |  |  |  |  |  |
| --- | --- | --- | --- | --- | --- | --- | --- | --- | --- | --- | --- |
| Compound | Scan type | Ionization | Precursor | Product | Fragment | Collision | Cell accelerator | Polarity | Injec. vol | Mobile phase | Flow (mL/min) |
|  |  | mode | ion (*m/z*) | ion (m/z) | voltage | Energy(V) | voltage |  |  |  |  |
| Ampicillin | MRM | ESI | 351 | 161.1 | 115 | 20 | 4 | positive | 5 ㎕ | A: 5 mM Ammonium formate + | 0.2 (A:B=50:50%) |
|  |  |  |  |  |  |  |  |  |  | 0.1% Formic acid in water |  |
|  |  |  |  | 107 |  | 20 |  |  |  | B: 5 mM Ammonium formate + |  |
|  |  |  |  |  |  |  |  |  |  | 0.1% Formic acid in methanol |  |
| Cefoperazone | MRM | ESI | 646 | 290 | 125 | 30 | 3 | positive | 1 ㎕ | A: 5 mM Ammonium formate + | 0.2 (A:B=30:70%) |
|  |  |  |  |  |  |  |  |  |  | 0.1% Formic acid in water |  |
|  |  |  |  | 143 |  | 30 |  |  |  | B: 5 mM Ammonium formate + |  |
|  |  |  |  |  |  |  |  |  |  | 0.1% Formic acid in methanol |  |
| Cefotoxime | MRM | ESI | 456 | 396 | 115 | 10 | 3 | positive | 1 ㎕ | A: 5 mM Ammonium formate + | 0.2 (A:B=50:50%) |
|  |  |  |  |  |  |  |  |  |  | 0.1% Formic acid in water |  |
|  |  |  |  | 324 |  | 10 |  |  |  | B: 5 mM Ammonium formate + |  |
|  |  |  |  |  |  |  |  |  |  | 0.1% Formic acid in methanol |  |

**SUPPLEMENTARY FIGURE LEGENDS**


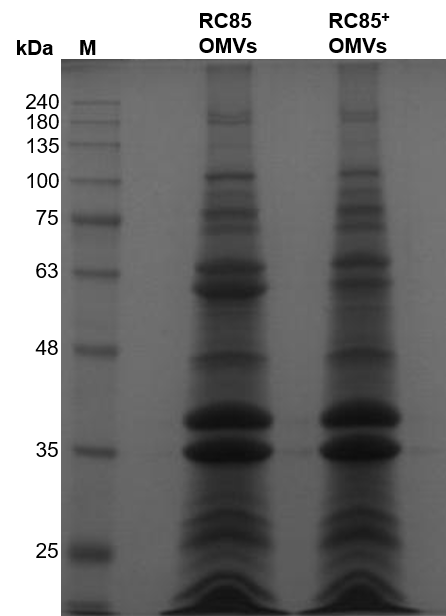


**Supplementary figure S1** Comparative proteomic profiles are shown for 60 μg of OMVs from RC85 and RC85^+^ cells resolved on 12% (w/v) SDS-PAGE and subjected to coomassie staining.
